# The Safety and Efficacy of Dexmedetomidine vs. Sufentanil in Monitored Anesthesia Care during Burr-Hole Surgery for Chronic Subdural Hematoma: A Retrospective Clinical Trial

**DOI:** 10.3389/fphar.2016.00410

**Published:** 2016-11-03

**Authors:** Wenming Wang, Lei Feng, Fenfen Bai, Zongwang Zhang, Yong Zhao, Chunguang Ren

**Affiliations:** ^1^Department of Neurosurgery, The First People's Hospital of Kunshan Affiliated with Jiangsu UniversitySuzhou, China; ^2^Department of Anesthesiology, Liaocheng People's HospitalLiaocheng, China

**Keywords:** dexmedetomidine, sufentanil, monitored anesthesia care, chronic subdural hematoma, burr-hole surgery

## Abstract

**Background:** Chronic subdural hematoma (CSDH) is a very common clinical emergency encountered in neurosurgery. While both general anesthesia (GA) and monitored anesthesia care (MAC) can be used during CSDH surgery, MAC is the preferred choice among surgeons. Further, while dexmedetomidine (DEX) is reportedly a safe and effective agent for many diagnostic and therapeutic procedures, there have been no trials to evaluate the safety and efficacy of DEX vs. sufentanil in CSDH surgery.

**Objective:** To evaluate the safety and efficacy of DEX vs. sufentanil in MAC during burr-hole surgery for CSDH.

**Methods:** In all, 215 fifteen patients underwent burr-hole surgery for CSDH with MAC and were divided into three groups: Group D1 (*n* = 67, DEX infusion at 0.5 μg·kg^−1^ for 10 min), Group D2 (*n* = 75, DEX infusion at 1 μg·kg^−1^ for 10 min), and Group S (*n* = 73, sufentanil infusion 0.3 μg·kg^−1^ for 10 min). Ramsay sedation scale (RSS) of all three groups was maintained at 3. Anesthesia onset time, total number of intraoperative patient movements, hemodynamics, total cumulative dose of DEX, time to first dose and amount of rescue midazolam or fentanyl, percentage of patients converted to alternative sedative or anesthetic therapy, postoperative recovery time, adverse events, and patient and surgeon satisfaction scores were recorded.

**Results:** The anesthesia onset time was significantly less in group D2 (17.36 ± 4.23 vs. 13.42 ± 2.12 vs. 15.98 ± 4.58 min, respectively, for D1, D2, S; *P* < 0.001). More patients in groups D1 and S required rescue midazolam to achieve RSS = 3 (74.63 vs. 42.67 vs. 71.23%, respectively, for D1, D2, S; *P* < 0.001). However, the total dose of rescue midazolam was significantly higher in group D1 (2.8 ± 0.3 vs. 1.9 ± 0.3 vs. 2.0 ± 0.4 mg, respectively, for D1, D2, S; *P* < 0.001). The time to first dose of rescue midazolam was significantly longer in group D2 (17.32 ± 4.47 vs. 23.56 ± 5.36 vs. 16.55 ± 4.91 min, respectively, for D1, D2, S; *P* < 0.001). Significantly fewer patients in groups S and D2 required rescue fentanyl to relieve pain (62.69 vs. 21.33 vs. 27.40%, respectively, for D1, D2, S; *P* < 0.001). Additionally, total dose of rescue fentanyl in group D1 group was significantly higher (212.5 ± 43.6 vs. 107.2 ± 35.9 vs. 98.6 ± 32.2 μg, respectively, for D1, D2, S; *P* < 0.001). Total number of patient movements during the burr-hole surgery was higher in groups D1 and S (47.76 vs. 20.00 vs. 47.95%, respectively, for D1, D2, S; *P* < 0.001). Four patients in D1 and five in S converted to propofol. The time to recovery for discharge from the PACU was significantly shorter in group D2 (16.24 ± 4.15 vs. 12.48 ± 3.29 vs. 15.91 ± 3.66 min, respectively, for D1, D2, S; *P* < 0.001). Results from the patient and surgeon satisfaction scores showed significant differences favoring group D2 (*P* < 0.05). More patients in groups D1 and S showed higher levels of the overall incidence of tachycardia and hypertension, and required higher doses of urapidil and esmolol (*P* < 0.05). Six patients experienced respiratory depression in group S.

**Conclusion:** Compared with sufentanil, DEX infusion at 1 μg·kg^−1^ was associated with fewer intraoperative patient movements, fewer rescue interventions, faster postoperative recovery, and better patient and surgeon satisfaction scores and could be safely and effectively used for MAC during burr-hole surgery for CSDH.

## Introduction

Chronic subdural hematoma (CSDH) usually occurs in the elderly with co-morbidities and multi-system disorders, and is one of the most frequently encountered intracranial hemorrhages in neurosurgery (Guzel et al., 2008; Ma and Fei, 2010). Burr-hole surgery is commonly used for its initial treatment(Xu et al., 2016). Previous studies have reported that general anesthesia (GA), local anesthesia, or monitored anesthesia care (MAC) alone could not provide adequate safety assurance for both patients and surgeons. Nowadays, more and more medical centers perform this procedure under local anesthesia associated with MAC for its safety and efficacy during the surgery (Cenic et al., 2005; Mekaj et al., 2008). Midazolam, propofol, opioids, or a combination of these drugs are widely used during MAC; however, each of these drugs has its limitations (Song et al., 2004; Agostoni et al., 2011; Sato et al., 2016). While midazolam has no analgesic property, its sedative pharmacological properties vary with different patients and may be responsible for respiratory depression. Propofol, which also lacks analgesic characteristics, is a short-acting anesthetic agent. As per the FDA guidelines, propofol administration should be closely monitored owing to easily induced respiratory depression and hemodynamic instability. Opioids such as fentanyl, sufentanil, and remifentanil can provide excellent analgesia without sedation. The incidence of adverse reactions, especially respiratory depression, will be increased when used in higher doses or in combination with other sedative agents. Most importantly, the American Society of Anesthesiologist's (ASA) Closed Claim Database revealed that respiratory depression without close monitoring played an important role in mishaps during MAC. (ASA Task Force on Sedation Analgesia by Non Anesthesiologists, 2002) Because of the unavoidable adverse effects of these drugs, the need for an ideal sedative agent with limited adverse effects that can be used safely and efficacy during MAC is urgent.

Dexmedetomidine (DEX), a highly selective agonist of the α2 adrenergic receptor, has a more favorable pharmacokinetic profile than clonidine: α2:α1 specificity ratio, 1600:1 vs. 200:1 and plasma half-life (T½), 2–2.5 h vs. 9–12 h. It also has many clinical benefits, such as sedation without significant respiratory depression, an analgesic-sparing effect, and a sympatholytic effect that can attenuate the stress response to surgery (Geloen et al., 2013). Previous studies have reported that DEX could be used safely and effectively for many diagnostic and therapeutic procedures (Jaakola et al., 1992; Demiraran et al., 2007). However, according to an independent search of MEDLINE, PubMed, EMBASE, Cochrane Central Register of Controlled Trials, and Web of Science for English language articles between 2000 and 2015 and using the terms “dexmedetomidine,” “sufentanil,” and “monitored anesthesia care,” there have been no trials that report the use of DEX vs. sufentanil for surgical treatment of CSDH. We conducted this retrospective trial to evaluate the safety and efficacy of DEX vs. sufentanil for MAC during burr-hole surgery for CSDH.

## Materials and methods

### Patients

The institutional review board of Liaocheng People's Hospital approved (No. 2016057) this retrospective trial, which was registered at chictr.org (ChiCTR-IPR-16008494). Patients who underwent burr-hole surgery for CSDH with MAC between January 2014 and December 2015 and provided written informed consent were enrolled in this study. The inclusion criteria were: age between 45 and 65 years and ASA grade I to III. Exclusion criteria included hypertension (diastolic blood pressure > 160 mmHg); bradycardia (<50 bpm); ischemic heart disease (<6 months); second- or third-degree heart block; long-term abuse of or addiction to alcohol, tobacco, opioids, and sedative–hypnotic drugs (>6 months); allergy to DEX and/or sufentanil; neuropsychiatric diseases; operation time shorter than 30 min; emergent surgery.

Patients were divided into the following three groups: Group D1 (*n* = 67, initial DEX infusion at 0.5 μg·kg^−1^ for 10 min, then adjusted to 0.2–0.7 μg·kg^−1^·h^−1^); Group D2 (*n* = 75, initial DEX infusion at 1 μg·kg^−1^ for 10 min, then adjusted to 0.2–0.7 μg·kg^−1^·h^−1^); and Group S (*n* = 73, initial sufentanil infusion at 0.3 μg·kg^−1^ for 10 min, then adjusted to 0.1–0.3 μg·kg^−1^·h^−1^). Electronic charting and DoCare Clinic electronic anesthesia recording system data were utilized during this trial. All patients received an explanation about the operative procedure, Ramsay sedation score (RSS), and pain score prior to the surgery. The burr-hole surgery for CSDH was performed by a neurosurgeon with ≥5 years of residency experience.

### Anesthesia management

No premedication was administered before surgery. Prior to starting the surgery, ASA standard monitoring five-lead electrocardiography, non-invasive arterial blood pressure, peripheral pulse-oximetry (SpO_2_), respiratory rate (RR), and temperature were continuously monitored using an automated system (Philips IntelliVue MP50). Oxygen supplementation at 3 L·min^−1^ was achieved through an oxygen mask; then, an 18-gauge intravenous catheter was placed in a peripheral vein under local infiltration anesthesia. A forced-air warming device (EQUATOR Convective Warmer, EQ-5000) was used during the surgery to maintain normothermia.

Patients in the D1 and D2 groups received an initial loading dose of 0.5 μg·kg^−1^ and 1 μg·kg^−1^ DEX, respectively, over 10 min followed by a maintenance infusion of 0.2–0.7 μg·kg^−1^·h^−1^ to achieve adequate sedation. Patients in group S received an initial loading dose of 0.3 μg·kg^−1^ sufentanil over 10 min followed by a maintenance infusion of 0.1–0.3 μg·kg^−1^·h^−1^ to achieve adequate sedation. The target RSS of the three groups was 3 (patient exhibits response to commands). A rescue bolus of midazolam 0.02 mg·kg^−1^ was repeated every 5 min to a maximum dose of 2.5 mg if RSS > 3 or movement was noted during the procedure, while fentanyl 1 μg·kg^−1^ was repeated every 5 min to a maximum dose of 0.2 mg if the pain score (visual analog scale, VAS) >4. If the patient did not reach the ideal status after the maximum dose of midazolam and fentanyl was reached, propofol or GA was administered. Once patients showed the ideal state of sedation, the scalp was infiltrated with 5 mL of a local anesthetic solution containing 2.5 mL of 0.5% hydrochloride ropivacaine and 2.5 mL of 2% lidocaine with adrenaline at each burr hole site. DEX and sufentanil infusion were stopped when the drainage tube was fixed. All patients received 5 mg of tropisetron and were transferred to the post-anesthesia care unit (PACU) after surgery.

On arrival at the PACU, vital signs (HR, non-invasive blood pressure, RR, SpO_2_, temperature), RSS, and VAS were monitored every 5 min for the first 20 min, then every 10 min for the rest of the time until the patients were discharged to the wards (Aldrete Score ≥ 9) (Mason et al., 2013). Surgeon satisfaction was assessed as follows 24 h after burr-hole surgery: 1, extremely dissatisfied; 2, not satisfied but able to manage; 3, satisfied; 4, extremely satisfied); patients were visited to assess satisfaction on a 7-point Likert verbal rating scale (Bagchi et al., 2014). Fentanyl, 1 μg·kg^−1^, was given repeated every 5 min to a maximum dose of 0.2 mg if VAS > 4; however, in patients with a poor response to fentanyl or in the event of an obvious fentanyl-associated adverse effect, 30 mg of ketorolac was administered.

During the surgery, bradycardia and tachycardia were defined as HR <45 bpm decrease or >120 bpm increase from baseline and treated with intravenous atropine, 0.2 mg, or esmolol, 0.4 mg·kg^−1^, respectively. Hypertension and hypotension were defined as a >20% increase or decrease from baseline and treated using urapidil (10–15 mg) or phenylephrine (20–80 μg), respectively. All patients were closely continuously monitored with the aid of five-lead electrocardiography and non-invasive arterial blood pressure, SpO2, and RR readings using an automated system (Philips IntelliVue MP50) for 48 h after surgery.

### Data collection

The intraoperative hemodynamic data (HR, non-invasive blood pressure, RR, SpO_2_, temperature) were obtained from a Philips IntelliVue monitor at the following time points: arrival at the operating room (T1); after bolus administration of drug (T2); before administration of local anesthetic (T3); before skin incision (T4); at 5 min (T5) and 10 min (T6) after skin incision; and at arrival (T7), 5 min (T8), and 10 min (T9) at the PACU. Anesthesia onset time (from the initiation of anesthesia induction to the onset of the surgical procedure), recovery time (from stopping the DEX or sufentanil infusion to achieve RSS = 2), total number of intraoperative patient movements, amount of rescue midazolam or fentanyl, time to first dose of rescue midazolam or fentanyl, percentage of patients converted to alternative sedative or anesthetic therapy, adverse events, and patient and surgeon satisfaction scores were recorded.

### Statistical analysis

The Kolmogorov–Smirnov test was used to assess the distribution of variables. Homogeneity of variance was determined using Levene's tests. Quantitative data were expressed as mean and standard deviation or median and inter-quartile range (IQR). Inter-group comparisons were performed using repeated-measures analysis of variance (ANOVA). The Bonferroni's correction was applied for *post-hoc* multiple comparisons. The non-parametric Kruskal–Wallis test was used for variables that were not normally distributed. Categorical data were expressed as frequency and percentage and analyzed using chi-squared tests or Fisher's exact tests when appropriate. Probability (*P*) values <0.05 were considered statistically significant. Statistical analysis was performed with SPSS for Windows Version 18.0 (SPSS Inc. Chicago, IL, USA).

## Results

### Baseline characteristics

**Figure 1:**
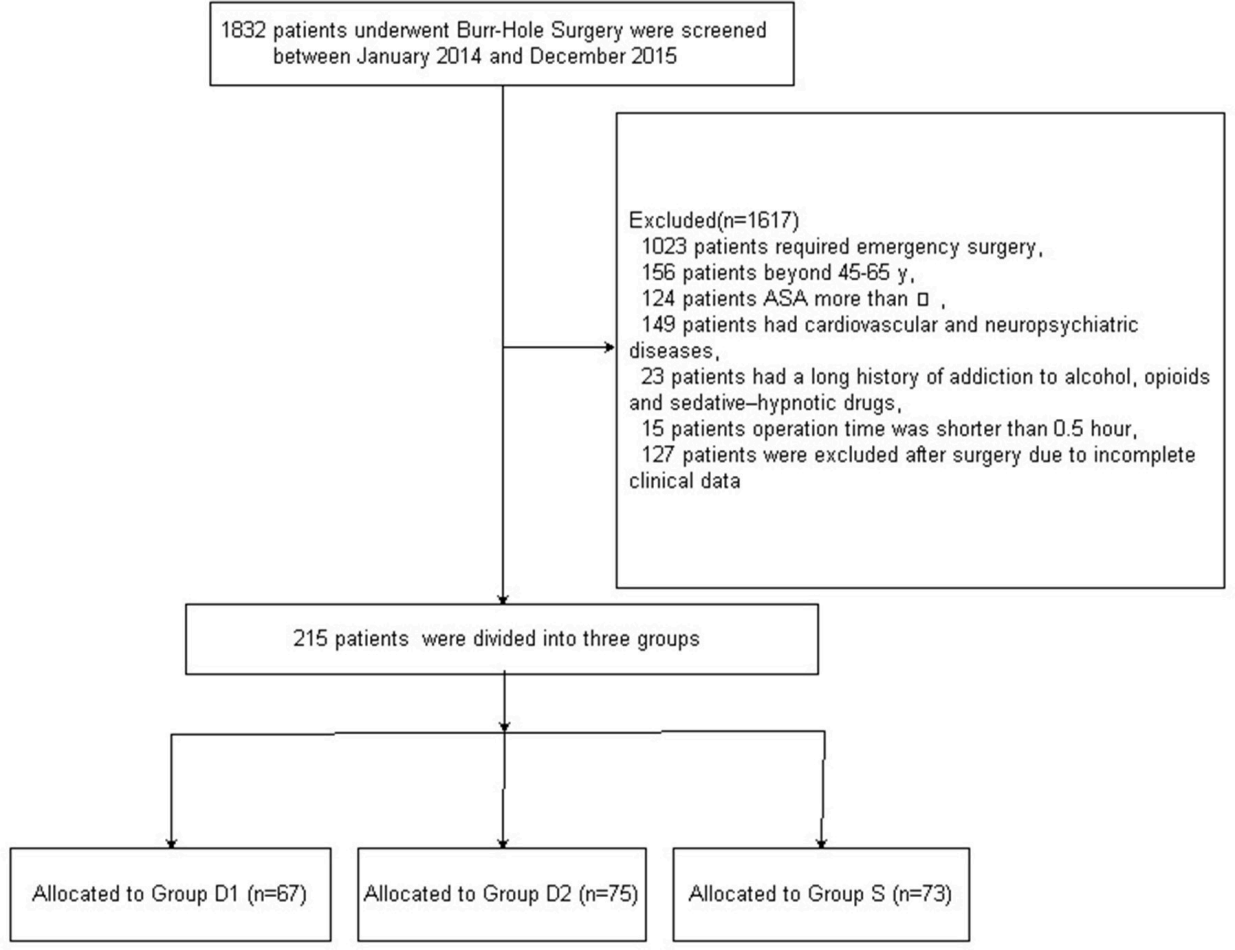
**Patient enrolment flow diagram**.

Figure 1 shows the CONSORT diagram of patient recruitment. Initially, 1832 patients who underwent burr-Hole surgery for CSDH were screened between January 2014 and December 2015. Of these, 1617 patients were excluded for not meeting the inclusion criteria: 1023 patients required emergency surgery; 156 patients did not fall into the specified age range of 45–65 years; the ASA grade of 124 patients was more than III; 149 patients had cardiovascular and neuropsychiatric diseases; 23 patients had a long history of addiction to alcohol, opioids, and sedative–hypnotic drugs; the operation time of 15 patients was shorter than 30 min; and 127 patients were excluded after surgery due to incomplete clinical data. Finally, 215 patients were included in the primary analysis. Demographic and baseline clinical parameters were not significantly different among the three groups (*P* > 0.05, Table 1).

**Table 1:** **Demographic and baseline clinical parameters in the three groups**.

**Variable**	**Group D1 (*n* = 67)**	**Group D2 (*n* = 75)**	**Group S (*n* = 73)**	***P*-values**
Age (y)	57.53 ± 9.33	59.02 ± 8.92	55.69 ± 7.92	0.070
Body weight (kg)	72.39 ± 8.43	69.83 ± 9.34	71.98 ± 7.20	0.144
Sex (male/female)	42/25	43/32	50/23	0.380
BMI (kg·m^−2^)	23.39 ± 3.12	24.02 ± 2.92	23.87 ± 2.78	0.419
ASA (I/II/III)	18/40/9	15/48/12	15/51/7	0.623
Preoperative GCS	14.78 ± 1.09	14.88 ± 0.92	14.82 ± 1.03	0.838
Hematoma volume (mL)	52.19 ± 12.29	56.29 ± 14.82	52.95 ± 15.37	0.187
Duration of anesthesia (min)	72.23 ± 14.24	78.29 ± 17.74	75.89 ± 18.24	0.104
Duration of surgery (min)	47.24 ± 9.83	51.29 ± 11.20	48.93 ± 11.06	0.080
Comorbidity, *n* (%)				0.991
Hypertension	45 (67.16%)	48 (64.00%)	42 (57.53%)	
Arrhythmia	8 (11.94%)	12 (16.00%)	11 (15.07%)	
Diabetes mellitus	9 (13.43%)	11 (14.67%)	8 (10.96%)	
COPD/asthma	3 (4.48%)	4 (5.33%)	2 (2.74%)	
Anemia	13 (19.40%)	16 (21.33%)	10 (13.70%)	

*Variables are presented as mean ± SD or number of patients n (%). BMI, body mass index; ASA, American Society of Anesthesiology; GCS, Glasgow Coma Scale; COPD, chronic obstructive pulmonary disease*.

### Intraoperative variables

**Figure 2:**
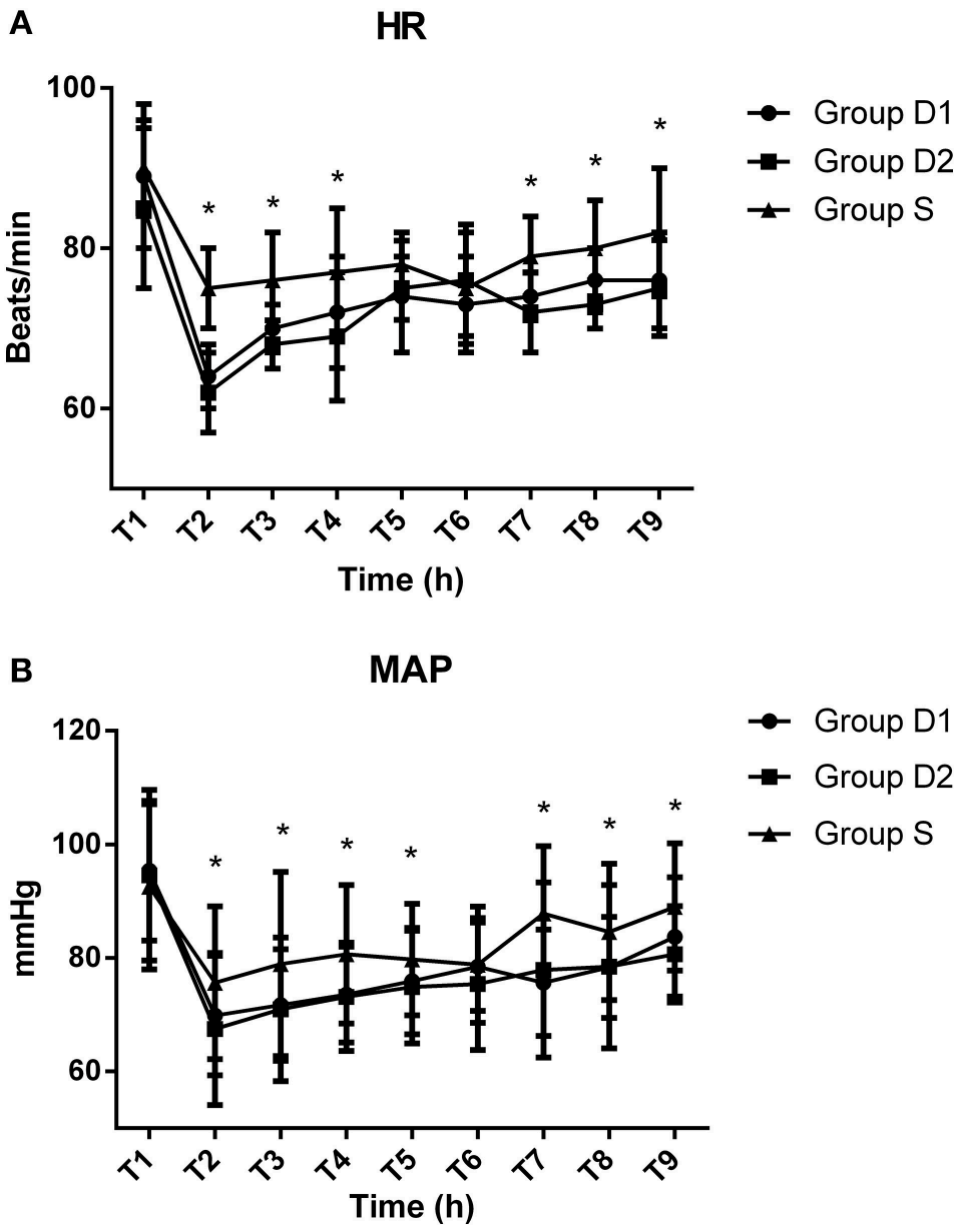
**Hemodynamics monitoring across the three groups**. **(A)** Comparison of heart rates (HR) (beats/min) among the three groups at different time points. **(B)** Comparison of mean arterial pressure (MAP) (mmHg) in the three groups at different time points. Baseline vital signs were not statistically difference among the three groups (*P* > 0.05). Compared with S group, both HR and MAP in the D1 and D2 groups were significantly decreased at T2 (after bolus administration of drug), T3 (before local anesthetic), T4 (before skin incision), T7 (arrival at the PACU), T8 (5 min after arrival at the PACU), and T9 (10 min after arrival at the PACU) (*P* < 0.05). The lowest levels of HR and MAP among the three groups both occurred at T2. ^*^P < 0.05 vs. group D2.

Baseline vital signs were not statistically different among the three groups (*P* > 0.05, Figure 2). Compared with S group, both D1 and D2 groups showed significantly decreased HR and MAP at T2, T3, T4, T7, T8, and T9 (*P* < 0.05, Figure 2). The lowest levels of HR and MAP among the three groups were recorded at T2.

**Figure 3:**
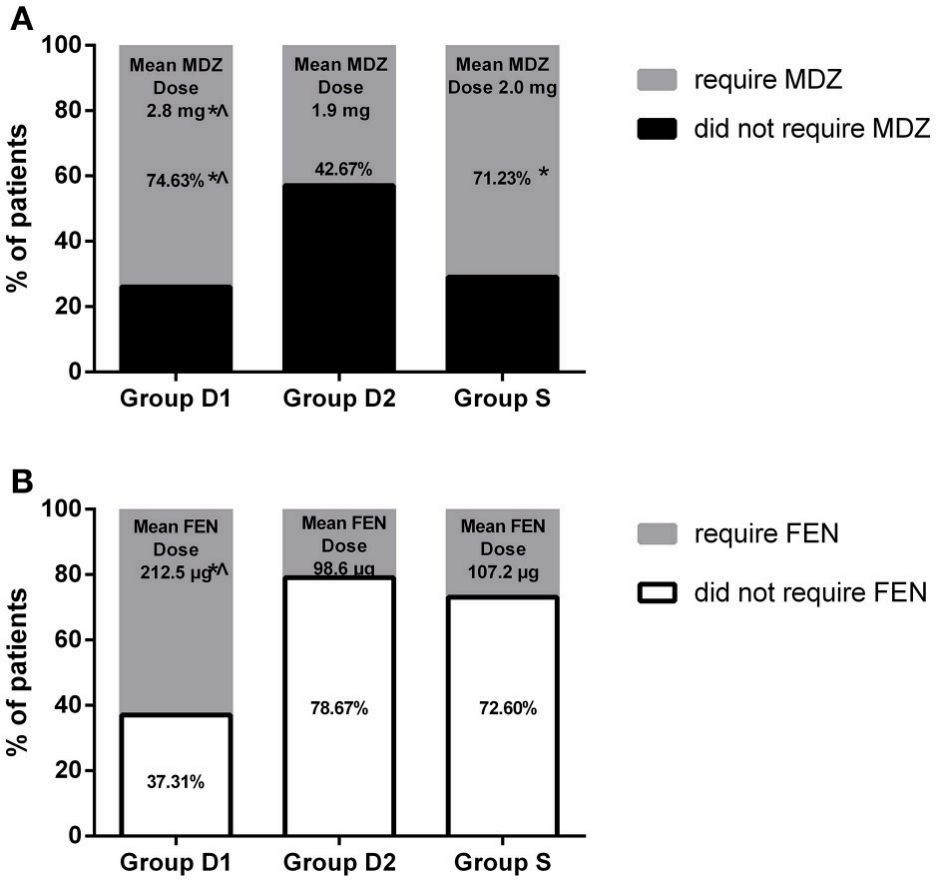
**(A)** Percentage of patients who required rescue midazolam, and mean midazolam dosage used during the study. **(B)** Percentage of patients who did not require rescue fentanyl, and mean fentanyl dosage used in patients requiring rescue fentanyl during the study. ^*^P < 0.05 vs. group D2, ^∧^*P* < 0.05 vs. group S.

Upon intergroup comparison, we found that anesthesia onset time was significantly shorter in group D2 (17.36 ± 4.23 vs. 13.42 ± 2.12 vs. 15.98 ± 4.58 min, respectively, for D1, D2, S; *P* < 0.001, Table 2), while durations of anesthesia and surgery and hematoma volume were not statistically different among the three groups (*P* > 0.05, Table 2). More patients in groups D1 and S required rescue midazolam to achieve RSS = 3 than in group D2 (74.63 vs. 42.67 vs. 71.23%, respectively, for D1, D2, S; *P* < 0.001, Figure 3A). However, the total dose of rescue midazolam was significantly higher in group D1 group than that in groups S and D2 (2.8 ± 0.3 vs. 1.9 ± 0.3 vs. 2.0 ± 0.4 mg, respectively, for D1, D2, S; *P* < 0.001, Figure 3A). The time to first dose of rescue midazolam was significantly longer in group D2 than in groups D1 and S (17.32 ± 4.47 vs. 23.56 ± 5.36 vs. 16.55 ± 4.91 min, respectively, for D1, D2, S; *P* < 0.001, Table 2). Significantly fewer patients in groups S and D2 required rescue fentanyl to relieve pain than in group D1 (62.69 vs. 21.33 vs. 27.40%, respectively, for D1, D2, S; *P* < 0.001, Figure 3B). Additionally, the total dose of rescue fentanyl in group D1 was significantly higher than that of groups S and D2 (212.5 ± 43.6 vs. 107.2 ± 35.9 vs. 98.6 ± 32.2 μg, respectively, for D1, D2, S; *P* < 0.001, Figure 3B). However, the time to first dose of rescue fentanyl was not statistically different among the three groups (18.47 ± 3.74 vs. 18.56 ± 2.92 vs. 18.24 ± 3.65 min, respectively, for D1, D2, S; *P* = 0.845, Table 2). Total cumulative dose of DEX was higher in group D2 (65.39 ± 18.53 vs. 92.15 ± 23.27 μg, respectively, for D1 and D2; *P* < 0.001, Table 2).

**Table 2:** **Comparison of intraoperative variables in the three groups**.

**Variable**	**Group D1 (*n* = 67)**	**Group D2 (*n* = 75)**	**Group S (*n* = 73)**	***P*-values**
Hematoma volume (mL)	52.19 ± 12.29	56.29 ± 14.82	50.95 ± 15.37	0.061
Duration of anesthesia (min)	72.23 ± 14.24	78.29 ± 17.74	75.89 ± 18.24	0.104
Duration of surgery (min)	47.24 ± 9.83	51.29 ± 11.20	48.93 ± 11.06	0.080
Anesthesia onset time (min)	17.36 ± 4.23	13.42 ± 2.12^*^^∧^	15.98 ± 4.58^*^	0.000
Time to first dose of rescue midazolam (min)	17.32 ± 4.47	23.56 ± 5.36^*^^∧^	16.55 ± 4.91	0.000
Time to first dose of rescue fentanyl (min)	18.47 ± 3.74	18.56 ± 2.92	18.24 ± 3.65	0.845
Total cumulative dose of dexmedetomidine (μg)	65.39 ± 18.53	92.15 ± 23.27^*^	0^*^^∧^	0.000
Total patient movements, *n* (%)	32 (47.76%)	15 (20.00%)^*^^∧^	35 (47.95%)	0.000
Converted to alternative sedative, *n* (%)	4 (5.97%)	0 (0.00%)^*^^∧^	5 (6.85%)	0.006

*Variables presented as mean ± SD or number of patients n (%)*.

* *P <0.05 vs. group D1*,

∧ *P <0.05 vs. group S*.

Total number of patient movements during the burr-hole surgery was higher in groups D1 and S (47.76 vs. 20.00 vs. 47.95%, respectively, for D1, D2, S; *P* = 0.000; Table 2). Although most of the patient movements could be controlled with reassurance, DEX or sufentanil infusion adjustment, and rescue drugs (midazolam or fentanyl), four patients from the D1 group and five from the S group still converted to an alternative sedative (propofol), while no patient required GA to complete the burr-hole surgery (Table 2).

### Postoperative variables

The time to recovery for discharge from the PACU (i.e., time to Aldrete score ≥9) was significantly shorter in group D2 than in groups S and D1 (16.24 ± 4.15 vs. 12.48 ± 3.29 vs. 15.91 ± 3.66 min, respectively, for D1, D2, S; *P* = 0.000, Table 3). Results from the patient and surgeon satisfaction scores showed significant differences favoring the D2 group compared with D1 and S groups (*P* < 0.05, Table 3).

**Table 3:** **Comparison of postoperative variables in the three groups**.

**Variable**	**Group D1 (*n* = 67)**	**Group D2 (*n* = 75)**	**Group S (*n* = 73)**	***P*-values**
Recovery time (min)	16.24 ± 4.15	12.48 ± 3.29^*^^∧^	15.91 ± 3.66	0.000
Patient satisfaction score	6.00 (5.25–7.00)	6.75 (5.75–7.00)^*^^∧^	6.25 (5.25–6.75)	0.035
Surgeon satisfaction score	1.75 (1.00–2.75)	3.25 (2.75–4.00)^*^^∧^	2.00 (1.25–3.00)	0.012

*Variables presented as mean ± SD or median (interquartile range)*.

* *P <0.05 vs. group D1*,

∧ *P <0.05 vs. group S*.

More patients in groups D1 and S than group D2 required higher doses of urapidil (41.79 vs. 20.00 vs. 34.25%, respectively, for D1, D2, S; *P* = 0.017, Table 4) and esmolol (32.84 vs. 16.00 vs. 31.51%, respectively, for D1, D2, S; *P* = 0.038, Table 4). The intraoperative dose of phenylephrine (11.94 vs. 12.00 vs. 13.70%, respectively, for D1, D2, S; *P* = 0.950, Table 4) and atropine (8.96 vs. 9.33 vs. 6.85%, respectively, for D1, D2, S; *P* = 0.824, Table 4) were comparable among the three groups during the surgery and in PACU.

**Table 4:** **Rescue drugs used in the three groups during surgery and in PACU**.

**Variable**	**Group D1 (*n* = 67)**	**Group D2 (*n* = 75)**	**Group S (*n* = 73)**	***P*-values**
Urapidil	28 (41.79%)	15 (20.00%)^*^^∧^	25 (34.25%)	0.017
Esmolol	22 (32.84%)	12 (16.00%)^*^^∧^	23 (31.51%)	0.038
Phenylephrine	8 (11.94%)	9 (12.00%)	10 (13.70%)	0.950
Atropine	6 (8.96%)	7 (9.33%)	5 (6.85%)	0.824

*Variables presented as number of patients n (%)*.

* *P <0.05 vs. group D1*,

∧ *P <0.05 vs. group S*.

The main adverse events are shown in Table 5. Patients in group D2 showed lower levels of the overall incidence of tachycardia (40.30 vs. 20.00 vs. 34.25% 25/73, respectively, D1, D2, S; *P* = 0.008, Table 5) and hypertension (44.78 vs. 22.67 vs. 36.99%, respectively, for D1, D2, S; *P* = 0.013, Table 5). The percentage of bradycardia, hypotension, nausea, and vomiting were comparable among the three groups during the surgery and in PACU (*P* > 0.05, Table 5). Six patients experienced respiratory depression (defined as a respiratory rate <8 times·min^−1^ or an oxygen saturation <90%) in group S.

**Table 5:** **Adverse events recorded in the three groups**.

	**Group D1 (*n* = 67)**	**Group D2 (*n* = 75)**	**Group S (*n* = 73)**	***P*-values**
Tachycardia	27 (40.30%)	15 (20.00%)^*^^∧^	25 (34.25%)	0.008
Hypertension	30 (44.78%)	17 (22.67%)^*^^∧^	27 (36.99%)	0.013
Bradycardia	8 (11.94%)	10 (13.33%)	7 (9.59%)	0.807
Hypotension	7 (10.45%)	11 (14.67%)	9 (12.33%)	0.745
Nausea	6 (8.96%)	5 (6.67%)	7 (9.59%)	0.797
Vomiting	2 (2.99%)	1 (1.33%)	2 (2.74%)	0.744
Respiratory depression	0 (0%)	0 (0%)^*^^∧^	6 (8.22%)^*^	0.004

*Variables presented as number of patients n (%)*.

* *P <0.05 vs. group D1*,

∧ *P <0.05 vs. group S*.

## Discussion

Compared with sufentanil, DEX infusion at 1.0 μg·kg^−1^ could decrease the number of intraoperative patient movements, which may be the primary reason for better patient and surgeon satisfaction scores found in the D2 group. In this study, we also found that the time to recovery from the PACU (i.e., time to Aldrete score ≥9) was significantly shorter in group D2 than in groups S and D1, likely because fewer patients in this group required rescue drugs. Four patients from group D1 and five from group S needed to convert to alternative sedation with propofol, although no patient required GA to complete the burr-hole surgery. Hypotension and bradycardia were the most common adverse events reported previously during the DEX infusion period; however, we did not find any significant differences among the three groups, and the few side effects seen were only mild or moderate in severity and responded well to intervention. Patients in group D2 also showed lower levels of tachycardia and hypertension. An important finding of our study was that a higher incidence of clinically significant respiratory depression was observed in the sufentanil group than in both DEX groups.

Monitored anesthesia care has been widely used in many clinical fields such as gastrointestinal endoscopy, septoplasty, thyroplasty, interventional or radiological procedures, cataract surgery, and awake bronchoscopy intubation (Bekker and Sturaitis, 2005; Busick et al., 2008; Goksu et al., 2008; Dogan et al., 2010; Parikh et al., 2013; Mondal et al., 2015). It can provide suitable intraoperative conditions for both patients and surgeons alike while avoiding the adverse reactions of GA, e.g., hemodynamic instability and prolonged emergence. Furthermore, the related hospitalization time and cost of medical expenses are both decreased compared to surgery under GA. The most commonly used drugs in MAC are midazolam; propofol; and opioids such as fentanyl, alfentanil, remifentanil, and sufentanil. Combining opioids with midazolam or propofol was the typical solution for patients with MAC in the past; however, increased side effects, especially cardio-respiratory depression, were being reported (Bhananker et al., 2006; Nonaka et al., 2015). Dexmedetomidine, a highly selective agonist of the α2 adrenergic receptor, acts as a sedative and anxiolytic agent through activation of the α2-adrenoreceptors located in the central nervous system. Unlike benzodiazepines and propofol, the sedation pathway of DEX does not depend on activation of the α-aminobutyric acid system (Bekker and Sturaitis, 2005). Besides, both spinal and supraspinal α2-adrenoreceptors may contribute to the analgesic-sparing and sympatholytic effects of DEX (Patel et al., 2010).

Intraoperative patient movement is one of the most common causes of complications during surgery, which is often because of inadequate sedation levels and analgesia. In this study, the incidence of total patient movements was least in the D2 groups and comparable in groups D1 and S. Our results are consistent with a previous study by Bekker et al. had reported. (Bekker et al., 2004) However, Muller et al. reported that DEX alone was not effective to provide sufficient sedation to complete the endoscopic retrograde cholangiopancreatography (ERCP). They also showed that patients in the DEX group were associated with greater hemodynamic instability and longer recovery time (Muller et al., 2008). Similarly, Tosun et al. found that among pediatric patients who underwent cardiac catheterization, sedation with DEX-ketamine was associated with insufficient sedation and longer recovery time than that with propofol-ketamine (Tosun et al., 2006). Recently, Koruk et al. stated that both DEX and ketamine in combination with propofol could be well tolerated in a small prospective randomized study of pediatric patients who underwent transcatheter atrial septal defect closure. However, the DEX-propofol group was associated with greater hemodynamic stability and shorter recovery time (Koruk et al., 2010). The inconsistent results among these studies may be attributed to different types of surgery, ways of anesthesia, or combination of drugs. In our study, the scalp was infiltrated with 5 mL of a local anesthetic solution containing 2.5 mL of 0.5% hydrochloride ropivacaine and 2.5 mL of 2% lidocaine with adrenaline at each burr hole site to reduce the dosage of opioids and alleviate patient discomfort. Therefore, the requirement for rescue drugs by the three groups in our study was less than that previously reported (Xu et al., 2014; Bishnoi et al., 2016; Surve et al., 2016). Although most patient movements could be controlled with reassurance, DEX or sufentanil infusion adjustment, or rescue drugs, nine patients still required to convert to alternative sedation with propofol, likely owing to the different mechanisms of DEX, sufentanil, midazolam, and propofol sedation (Payen et al., 2007; Lebherz-Eichinger et al., 2016).

In our study, the patient satisfaction score was higher in group D2 than in groups D1 and S. Patient satisfaction is a direct measure of the anesthetic care quality during surgery. A similar result was reported by Alhashemi, who attributed this finding to the analgesic property of DEX (Alhashemi, 2006). At the same time, we recorded the surgeon satisfaction score, which favored group D2. This may possibly be because of fewer intraoperative patient movements in the D2 group (Parikh et al., 2013).

Previous studies have reported that higher concentrations of DEX can result in fatal adverse effects, especially by affecting the cardiovascular system (Ebert et al., 2000; Gerlach and Murphy, 2009). However, consistent with the report of Dere et al. (2010), we found that more patients in groups D1 and S required higher doses of urapidil and esmolol, while the doses of phenylephrine and atropine were comparable among the three groups during the surgery and in the PACU. We believe this could be attributed to the sympathomimetic action of DEX (Dere et al., 2010). Further, six patients in our study experienced respiratory depression in group S, but none of them required conversion to GA. The unique sedative property of DEX may be the reason for the observed differences among the three groups in our study. Unlike in previously reported studies, the percentage of bradycardia, hypotension, nausea, and vomiting were comparable among the three groups during the surgery and in the PACU; this could be because of the shorter observation time, different types of surgery and patients, and mode of anesthesia administration (Muttu et al., 2005; Jessen Lundorf et al., 2016; Song et al., 2016).

We acknowledge that our study has some limitations. First, this study was designed as a retrospective trial, while a multi-center prospective design would have been more appropriate to verify the feasibility of DEX alone used during burr-hole surgery for CSDH. Second, although DEX was administered at a rate of 1.0 μg·kg^−1^ for 10 min, and then continued at a rate of 0.2–0.7 μg·kg^−1^·h^−1^ throughout the duration of the operation, we were unable to measure the serum concentration of DEX owing to technical limitations and increasing hospital costs. Third, six patients experienced respiratory depression in group S, which may be due to the high dose of sufentanil used in this study. Lastly, patients treated with beta-blockers and clonidine before surgery who developed bradycardia (<50 bpm) during the loading dose of DEX may not be suitable for this mode of anesthetic administration. In future, more studies should be carried out to verify the feasibility of different doses of sufentanil alone, or in combination with other drugs, in burr-hole surgery.

In summary, our study showed that compared with sufentanil, DEX infusion at 1 μg·kg^−1^ for 10 min (and subsequently adjusted to 0.2–0.7 μg·kg^−1^·h^−1^) alone could decrease the number of intraoperative patient movements and amount of rescue scheme, shorten postoperative recovery time, and provide better patient and surgeon satisfaction. At the same time, no severe adverse effects were recorded in either DEX group. Thus, DEX at the studied doses is a safe and effective primary sedation alternative to traditional opioids in patients undergoing MAC for burr-hole surgery.

## Author contributions

YZ, CR, and WW conceived and designed the trial; LF and WW collected the data; WW and FB analyzed the data; and ZZ, YZ, CR, and WW wrote this paper. YZ and CR contributed equally to this trial and considered as corresponding authors.

### Conflict of interest statement

The authors declare that the research was conducted in the absence of any commercial or financial relationships that could be construed as a potential conflict of interest.
